# A Humanized Anti‐gD Broadly Neutralizing Antibody Confers Complete Post‐Exposure Protection against Pseudorabies Virus

**DOI:** 10.1002/advs.75771

**Published:** 2026-05-19

**Authors:** Yue Sun, Jianbo Liu, Shi‐Jia Xu, Meng‐Xin Wang, Hongliang Zhang, Yanhe Zhang, Zhi‐Jun Tian, Jin‐Mei Peng, Xin Yin, Tong‐Qing An, Xue‐Hui Cai, Yan‐Dong Tang

**Affiliations:** ^1^ State Key Laboratory of Animal Disease Control and Prevention Harbin Veterinary Research Institute of Chinese Academy of Agricultural Sciences Harbin China; ^2^ Heilongjiang Provincial Research Center for Veterinary Biomedicine Harbin Veterinary Research Institute of Chinese Academy of Agricultural Sciences Harbin China; ^3^ Heilongjiang Provincial Key Laboratory of Veterinary Immunology Harbin Veterinary Research Institute of Chinese Academy of Agricultural Sciences Harbin China

**Keywords:** glycoprotein D, humanized antibody, neutralizing antibody, pseudorabies virus

## Abstract

Pseudorabies virus (PRV), an α‑herpesvirus, has recently been recognized as a potentially significant zoonotic pathogen, causing severe encephalitis and endophthalmitis in humans with high mortality and disability rates. Despite its growing public‑health threat, no effective therapeutics are available for human PRV infection. Here, we isolated a broadly neutralizing monoclonal antibody, designated 6F7, which targets the PRV glycoprotein D (gD) and neutralizes all tested PRV lineages. Mechanistic studies reveal that 6F7 does not impede viral attachment or internalization; instead, it specifically blocks the fusion of the viral envelope with cellular membranes, thereby preventing viral replication. Moreover, the antibody also potently suppresses cell‑to‑cell spread of PRV. We demonstrate that the interaction between gD and the PRV receptor Nectin‑1 is blocked by 6F7. Last, a humanized version of 6F7 confers complete protection in mice challenged with a lethal PRV variant and blocks the establishment of latency at a dose of 15 mg/kg per mouse. Overall, the humanized broadly neutralizing antibody represents a promising therapeutic candidate for human PRV infection.

## Graphical abstract

Pseudorabies virus (PRV), an emerging zoonotic α‐herpesvirus, causes life‐threatening human encephalitis. We identified a broad‐spectrum neutralizing antibody 6F7 targeting PRV gD. It blocks gD‐Nectin‐1 binding and membrane fusion, inhibiting replication across all PRV variants, representing a promising candidate for anti‐PRV therapy.

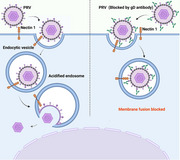

## 1 Introduction

Pseudorabies virus (PRV), an alphaherpesvirus and the causative agent of Aujeszky's disease, has historically been associated with substantial economic losses in global swine production [1]. However, PRV has undergone a significant epidemiological shift in recent years, emerging as a potential zoonotic pathogen capable of causing severe and often fatal encephalitis and endophthalmitis in humans [2, 3]. This paradigm shift underscores a growing and underestimated public health threat.

The genetic diversity of PRV is characterized by two major genotypes [2, 4]. Genotype I, historically endemic in Europe and North America, has been largely eliminated from commercial swine herds in these regions [2]. In contrast, Genotype II is now the dominant lineage in China and comprises two distinct clades: classical strains, prevalent before 2011, and variant strains responsible for outbreaks since 2011 [4]. Critically, recent human infections in China are almost exclusively associated with the Genotype II variant strains, highlighting their heightened zoonotic potential [3, 5].

Reported cases of human PRV infection have risen markedly, with approximately 49 documented cases to date, including 31 laboratory‐confirmed infections [2]. The clinical outcomes are severe: among confirmed cases, the mortality rate reaches 16.13% (5/31) [2]. Survivors often face devastating long‐term neurological sequelae, including cognitive impairment (42.31%, 11/26), motor disabilities (15.38%, 4/26), memory deficits (7.69%, 2/26), and epilepsy (7.69%, 2/26) [2]. Ocular complications are notably frequent and severe, affecting 57.69% (15/26) of survivors and frequently resulting in irreversible vision loss [2]. Despite comprehensive therapeutic interventions—including antiviral agents (e.g., acyclovir, ganciclovir), corticosteroids, and immunoglobulins—clinical outcomes remain poor, underscoring the critical need for effective therapeutics [2].

To date, no antiviral drugs have received regulatory approval for human PRV infection. While animal vaccines provide broad protection and are essential for preventing animal‑to‑human transmission, infected individuals lack specific pharmacologic interventions [2]. Monoclonal antibodies (mAbs) represent a promising antiviral strategy, offering unique advantages in post‑exposure prophylaxis, treatment of immunocompromised patients, and personalized medicine. Moreover, mAbs can target highly conserved epitopes, conferring protection against emerging variants—a concept validated by the success of anti‑SARS‑CoV‑2 and anti‐Chikungunya virus antibodies in recent years [6, 7].

Therefore, it is crucial to identify PRV‐specific neutralizing antibodies and elucidate their epitopes and mechanisms of action. Notably, several neutralizing antibodies have been developed against other herpesviruses [8, 9, 10, 11]. The PRV envelope glycoprotein D (gD) is essential for viral entry, mediating interaction with host receptors such as Nectin‑1 and triggering virus entry [12, 13, 14]. Therefore, gD is an attractive target for neutralizing antibodies and serological detection [15, 16]. Although several anti‑PRV neutralizing antibodies have been reported, they mostly target viral glycoprotein B (gB) and rely on complement‑mediated neutralization [14, 17]. While gD‐directed antibodies have been described, their virus specificity, molecular mechanism, and translational potential for humanization remain poorly defined [18, 19].

Here, we report the isolation and functional characterization of a monoclonal antibody, designated 6F7, which potently neutralizes all tested PRV lineages by uniquely inhibiting virus‑cell membrane fusion and viral cell‐to‐cell spread. We subsequently humanized 6F7 and demonstrated its robust efficacy in a post‑exposure therapeutic mouse model. Our findings not only introduce a promising clinical candidate for the treatment of human PRV infection but also provide mechanistic insights that can guide antibody development against other neurotropic herpesviruses.

## 2 Results

### 2.1 Isolation and Characterization of a Neutralizing Antibody Against PRV

**FIGURE 1:**
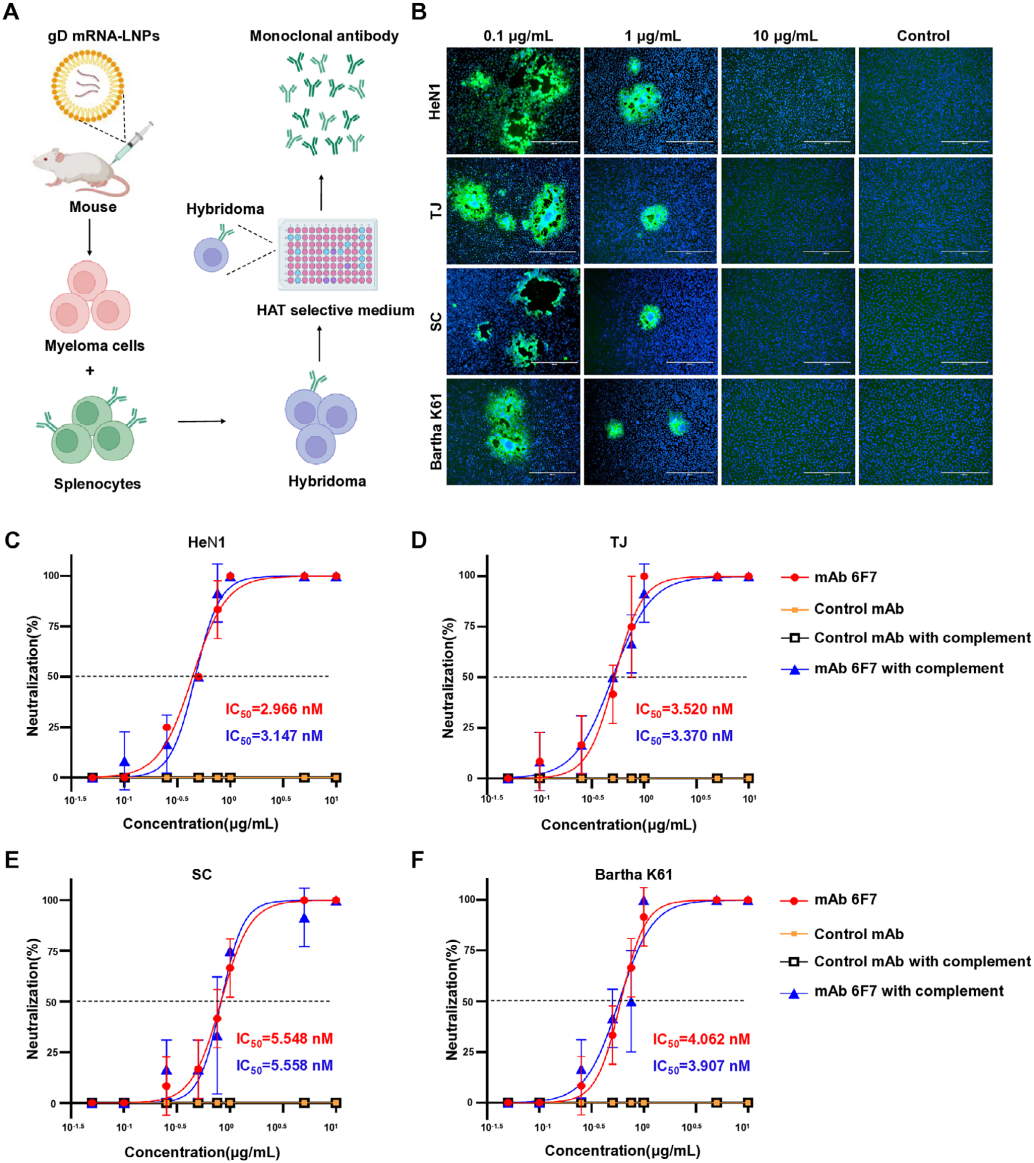
Neutralizing activity of mAb 6F7 against various PRV strains. (A) Schematic diagram of monoclonal preparation. (B) Neutralization of diverse PRV strains by mAb 6F7 without complement. Indicated concentrations of mAb 6F7 were incubated with 200 TCID_50_ of PRV strains (HeN1, TJ, SC, or Bartha K61) for 2 h at 37°C. The mixtures were then applied to Vero‐E6 cells for 2 h at 37°C. After removal of the inoculum, cells were washed and cultured in fresh medium containing 2% FBS. At 48 h post‐infection, infected cells were detected by immunofluorescence assay (IFA) targeting the PRV gB protein. The experiment was repeated three times, and a representative image is shown. Scale bars, 400 µm. (C) Determination of the half‐maximal inhibitory concentration (IC_50_) of mAb 6F7 in the presence (blue line) and absence (red line) of complement against the PRV HeN1, (D) TJ, (E) SC, and (F) Bartha K61 strains using a neutralization assay. 6F7 were heat‐inactivated at 56°C to remove the effect of complement. And in order to add complement, 6F7 were added 5% fresh mouse serum after heat‐inactivation. The experiment was repeated three times, and the results are presented as mean ± SD. IC_50_ values were calculated using GraphPad Prism 8.0 software.

To obtain neutralizing antibodies against PRV, we immunized mice with a gD‑encoding mRNA vaccine encapsulated in lipid nanoparticles (LNPs), and the immunization schedule is shown in Figure 1A [15]. After three rounds of gD mRNA‑LNPs immunizations, splenic B cells were harvested and fused with the mouse myeloma cell line SP2/0. Then, the viral inhibition assays were performed using the supernatant of hybridoma cells, and clone 6F7 showed the strongest inhibitory activity against the virus. Indirect immunofluorescence assays (IFA) demonstrated that 6F7 binds to representative strains from different PRV genotypes: the genotype II variant strains HeN1 and TJ, the genotype II classic strain SC, and the genotype I reference strain Bartha K61 (Figure S1). These results indicate that the epitope recognized by 6F7 is highly conserved across PRV lineages, conferring broad cross‑reactivity. In vitro neutralization assays showed that at a concentration of 10 µg/mL, 6F7 completely neutralized all tested PRV genotypes, preventing the appearance of virus‑induced cytopathic effects (Figure 1B). The half‑maximal inhibitory concentrations (IC_50_) for each strain were subsequently determined. These data demonstrate that 6F7 exhibits exceptional inhibitory activity against all known PRV lineages, with the strongest neutralization observed against the HeN1 variant (IC_50_  =  2.966 nm) (Figure 1C). The IC_50_ values for the other strains are all below 5.548 nm, confirming the antibody's broad‑spectrum neutralizing capability (Figure 1C–F). To dissect the relationship between the antiviral activity of 6F7 and complement, we further performed complement‐supplemented experiments, which demonstrated that the presence or absence of complement did not exert a significant impact on its antiviral activity (Figure 1C–F). In summary, we have successfully isolated a monoclonal antibody, 6F7, that cross‐neutralizes multiple PRV genotypes and whose antiviral activity is independent of complement, thereby providing a solid experimental foundation for its further development as a therapeutic candidate.

### 2.2 Prediction and Identification of the Neutralizing Epitope

**FIGURE 2:**
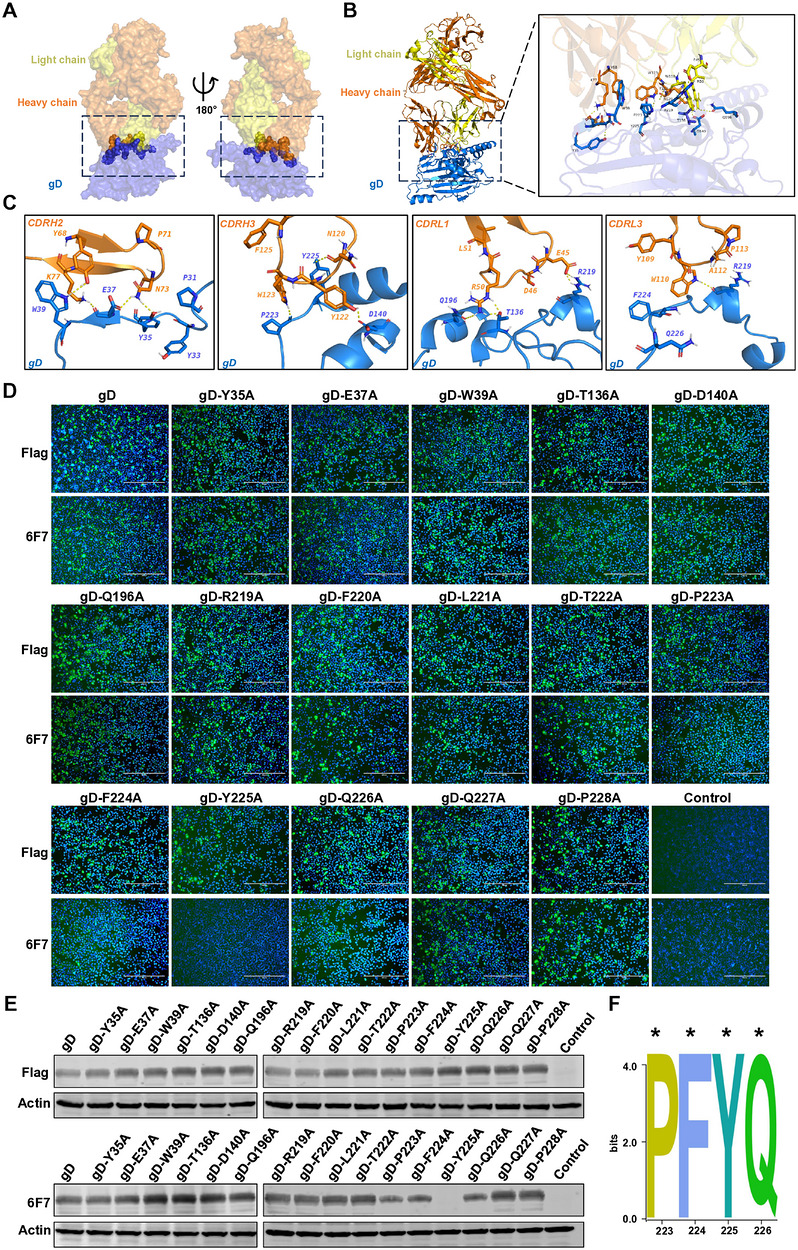
Identification of the mAb 6F7 binding epitope on PRV gD. (A) Surface representation of the mAb 6F7/PRV gD complex structure. PRV gD is colored blue. The heavy and light chains of mAb 6F7 are shown in orange and yellow, respectively. The interaction interface is highlighted with spheres. (B) Cartoon representation of the overall structure of the mAb 6F7/PRV gD complex. Color scheme is identical to (A). (C) Detailed view of the interactions between mAb 6F7 and PRV gD. Hydrogen bonds are indicated by yellow dashed lines. PRV gD is shown in blue. Complementary‐determining regions CDRH2 and CDRH3 (heavy chain), and CDRL1 and CDRL3 (light chain) are depicted in orange. All panels show the same orientation. (D) Functional assessment of key gD residues involved in mAb 6F7 binding. HeLa cells were transfected with wild‐type or mutant PRV gD constructs containing the indicated point mutations and analyzed by IFA for mAb 6F7 reactivity. The experiment was repeated three times, and a representative image is shown. (E) Western blotting analysis of gD constructs containing the indicated point mutations with mAb 6F7. The experiment was repeated three times, and a representative image is shown. (F) Conservation analysis of the mAb 6F7 epitope across PRV strains. A total of 38 gD amino acid sequences (4 genotype I, 5 genotype II classic, and 29 genotype II variant strains) from GenBank were analyzed for residue conservation within the binding region using TBtools II software.

To delineate the epitope recognized by 6F7, we first employed AlphaFold 3 to generate a structural model of the antibody‐gD complex [20]. The antibody heavy‑ and light‑chain sequences together with the PRV gD sequence were submitted to the server. The resulting surface representation of the predicted 6F7/gD complex clearly shows that the antibody can engage the gD protein (Figure 2A). A cartoon representation of the overall architecture of the complex further illustrates the relative orientation of the 6F7 and gD (Figure 2B). To examine the putative contact residues in detail, we visualized the interaction interface (Figure 2C). The model predicts nine potential contact sites on gD that form hydrogen‑bond networks with the complementarity‑determining regions (CDR) of 6F7. Each of these nine residues was subjected to site‑directed mutagenesis followed by functional assessment of antibody binding (Figure 2D). Substitution of Pro 223 with alanine (P223A) caused a marked reduction in 6F7 reactivity, whereas the Y225A mutation completely abolished antibody binding, indicating that residues P223 and Y225 constitute critical determinants of the 6F7 epitope (Figure 2D). Western blotting analysis via SDS‐PAGE demonstrated that 6F7 could recognize denatured proteins, confirming that 6F7 targets a linear epitope (Figure 2E). We therefore performed site‐directed mutagenesis of epitope residues adjacent to P223 and Y225, which revealed that F224 and Q226 are also crucial for 6F7 recognition (Figure 2D,E). To evaluate the conservation of the identified epitope across PRV isolates, we performed a conservation analysis using TBtools II [21]. A total of 38 gD amino‑acid sequences (4 genotype I, 5 genotype II classic, and 29 genotype II variant strains) retrieved from GenBank were aligned [16]. The analysis revealed that P223, F224, Y225, and Q226 are highly conserved among the examined PRV strains (Figure 2F). Collectively, these data define a conserved linear epitope on PRV gD centered on residues P223, F224, Y225, and Q226 that is essential for 6F7 binding and neutralization.

### 2.3 6F7 Neutralizes PRV Without Inhibiting Virus‑Cell Attachment

**FIGURE 3:**
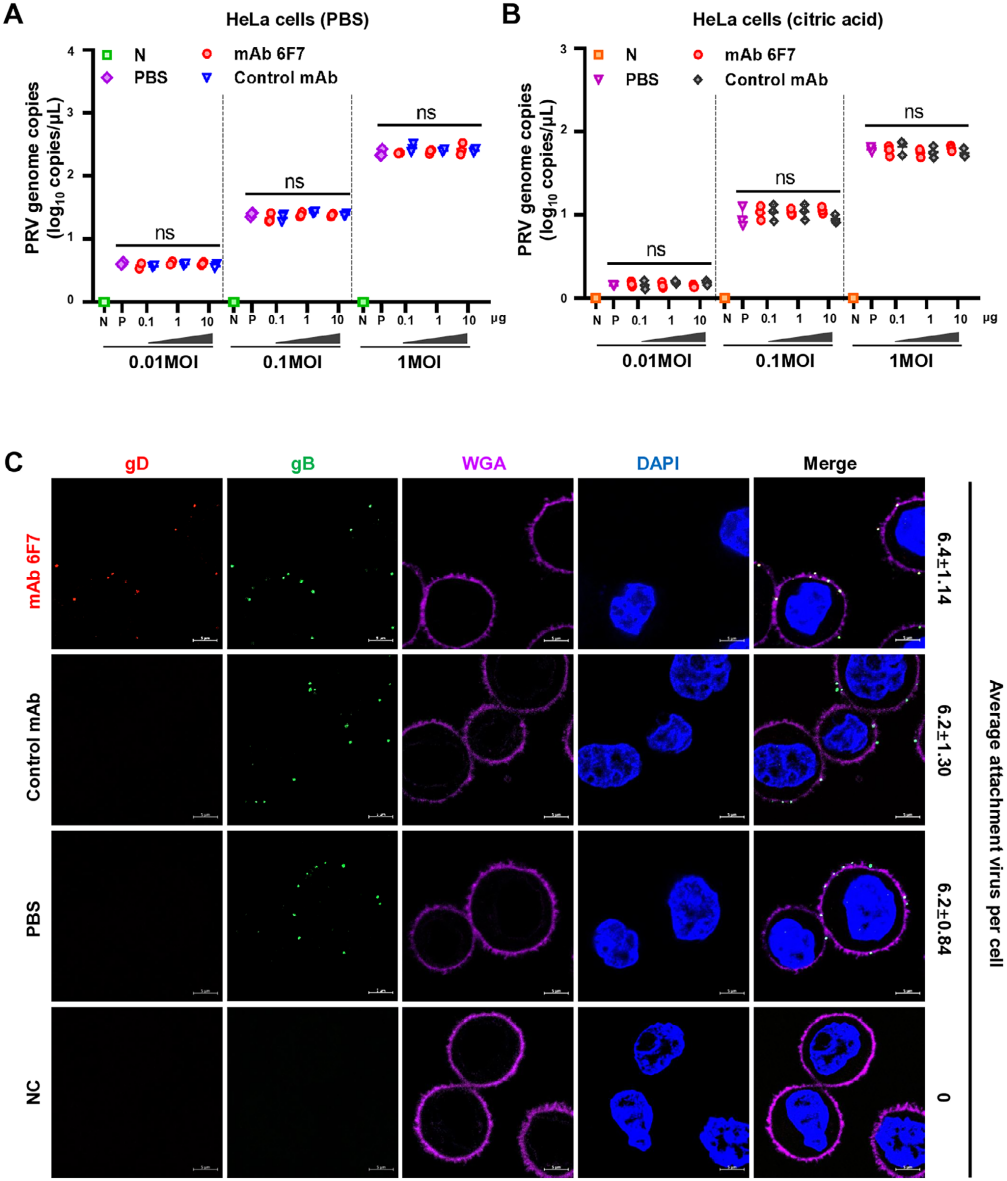
mAb 6F7 does not inhibit PRV attachment to HeLa cells. (A) Quantification of cell‐associated viral DNA following attachment. mAb 6F7 or an isotype control antibody (0.1 µg, 1 µg, or 10 µg) was pre‐incubated with PRV HeN1 (at MOIs of 0.01, 0.1, or 1) for 2 h at 37°C. The mixtures were then added to pre‐chilled HeLa cells and incubated at 4°C for 2 h to allow attachment. Unbound virus was removed by washing with PBS or (B) citric acid, and cell‐associated viral DNA was quantified by qRT‐PCR. The experiment was repeated three times, and the results are presented as mean ± SD. (C) Visualization of attached viral particles by confocal microscopy. The mAb 6F7 or control mAb (50 µg) was mixed with PRV HeN1 (MOI = 10) and incubated for 2 h at 37°C. The mixture was applied to pre‐chilled HeLa cells in 24‐well plates for 2 h at 4°C. After washing, cells were fixed, blocked, and stained with an Alexa Fluor 568‐conjugated anti‐mouse IgG (to detect bound mAb 6F7, red), FITC‐conjugated anti‐gB mAb (green), WGA (to stain the cell membrane, violet), and DAPI (blue). The experiment was repeated three times, and a representative image is shown. The average virus per cell was quantified. Scale bars, 5 µm.

The PRV life cycle can be divided into the following key steps: attachment to the cell surface, internalization, virus‑cell membrane fusion, followed by viral genome replication, assembly, and release from infected cells. To determine whether the neutralizing activity of 6F7 is mediated by blocking the attachment step, we first examined virus adsorption in the presence of increasing concentrations of the antibody. HeLa cells were inoculated with the mixtures of different doses of the virus (MOI = 0.01, 0.1, and 1) and indicated dose of antibody at 4°C; after 2 h, the cells were washed with PBS, and we found that viral attachment was not affected by the antibody (Figure 3A). To rule out the interference of non‐specific adsorption, we included a parallel control group in which HeLa cells were pretreated with citric acid. We observed that, although viral adsorption was reduced relative to the PBS‐washed control group, the antibody still had no effect on viral adsorption in the citric acid‐treated group (Figure 3B). Consistent results were obtained in PK15 cells, Vero‐E6 cells, and HEK293T cells (Figure S2). For a more direct visual confirmation, we employed laser‑scanning confocal microscopy. PRV HeN1 (MOI  =  10) was pre‑incubated with either 6F7 or a control mAb (50 µg) for 2 h at 37°C, then added to pre‑chilled HeLa cells and allowed to bind for 2 h at 4°C. After washing, cells were fixed, blocked, and stained with an Alexa Fluor 568‑conjugated anti‑mouse IgG (red, to detect bound antibody) and a FITC‑conjugated anti‑gB monoclonal antibody (green, to label virions) (Figure S3). Confocal images demonstrated that both 6F7 and the control antibody failed to prevent PRV particles from attaching to the cell surface (Figure 3C). Collectively, the results from these different cell lines showed that 6F7 did not impede viral attachment to any of the cell types tested.

### 2.4 6F7 Does Not Interfere with PRV Internalization

**FIGURE 4:**
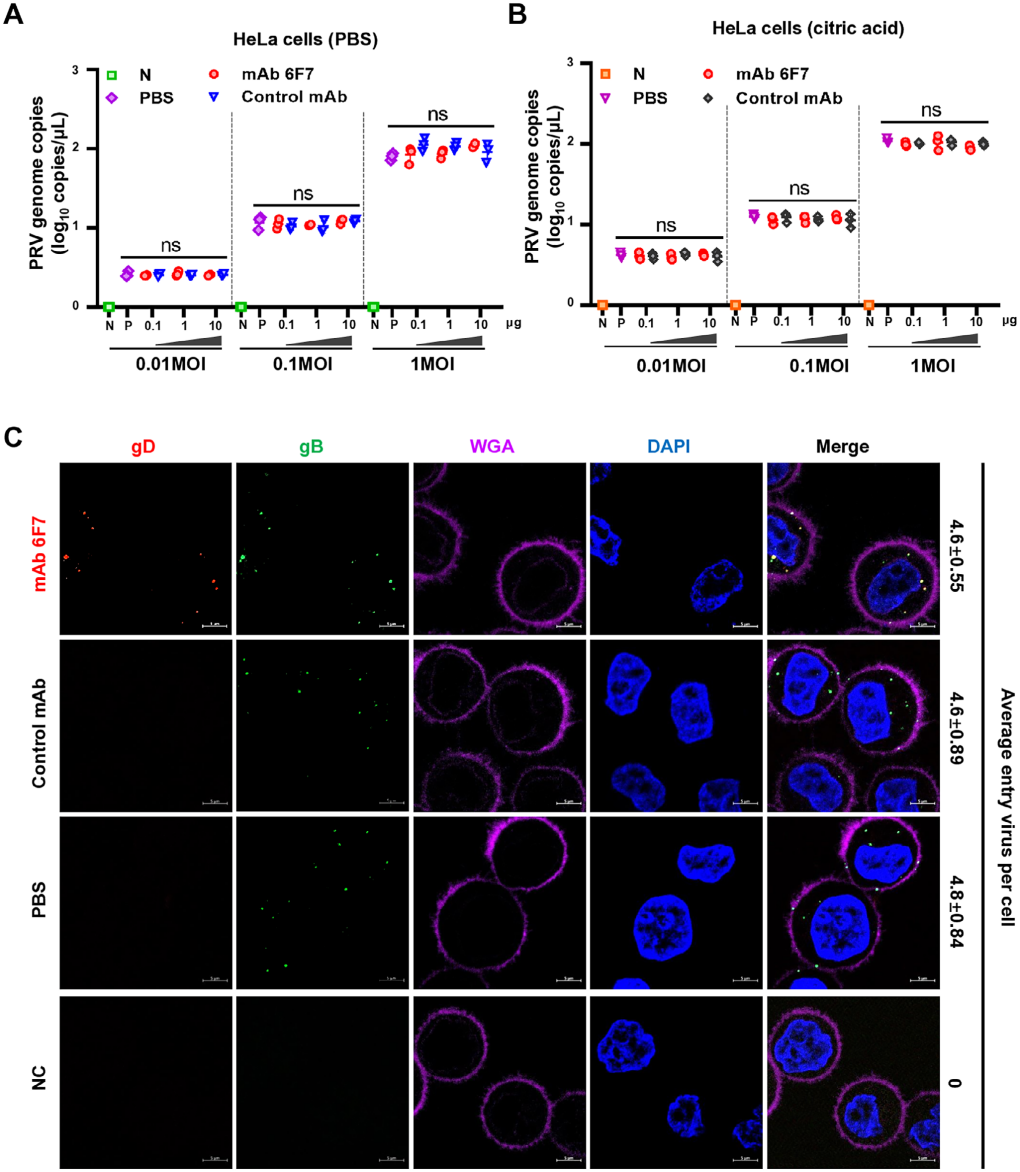
mAb 6F7 does not affect PRV internalization in HeLa cells. (A) Quantification of internalized viral DNA. The mAb 6F7 or control mAb (0.1, 1, or 10 µg) was pre‐incubated with PRV HeN1 (at MOIs of 0.01, 0.1, or 1) for 2 h at 37°C. Mixtures were added to pre‐chilled HeLa cells and incubated at 4°C for 2 h for attachment. The inoculum was then removed, cells were washed with PBS or (B) citric acid, fresh pre‐warmed medium was added, and cells were shifted to 37°C for 2 h to permit internalization. After washing, cells were harvested, and internalized viral DNA was quantified by qRT‐PCR. The experiment was repeated three times, and the results are presented as mean ± SD. (C) Visualization of internalized viral particles by confocal microscopy. The mAb 6F7 or control mAb (50 µg) was mixed with PRV HeN1 (MOI = 10) and incubated for 2 h at 37°C. The mixture was applied to pre‐chilled HeLa cells for 2 h at 4°C. Unbound virus was removed, fresh medium was added, and cells were incubated at 37°C for 2 h. Cells were then fixed, permeabilized, blocked, and stained. The experiment was repeated three times, and a representative image is shown. The average virus per cell was quantified. Scale bars, 5 µm.

Due to that 6F7 does not block viral attachment, we next examined whether the antibody impeded the subsequent internalization step. HeLa cells were infected with PRV that had been pre‑incubated with a titration series of 6F7 or with an isotype‑matched control mAb. After a 2 h adsorption period at 4°C to allow binding without entry, the inoculum was removed by PBS or citric acid, cells were shifted to 37°C to trigger internalization, and the reaction was terminated at 2 h post‑temperature shift. The amount of internalized viral DNA was quantified, and the results indicated that the level of internalized virus was indistinguishable between 6F7‐treated and control samples (Figure 4A,B). Consistent results were also obtained in PK15, Vero‐E6, and HEK293T cells (Figure S4). To corroborate these findings using microscopy, we performed a internalization assay coupled with confocal imaging. We observed a comparable number of intracellular fluorescent puncta in both the 6F7‐treated and control groups (Figure 4C). Collectively, these data demonstrate that 6F7 does not block PRV internalization following attachment, indicating that its neutralizing activity acts downstream of virus internalization.

### 2.5 6F7 Neutralizes PRV by Blocking Viral‑Envelope–Cell‑Membrane Fusion

**FIGURE 5:**
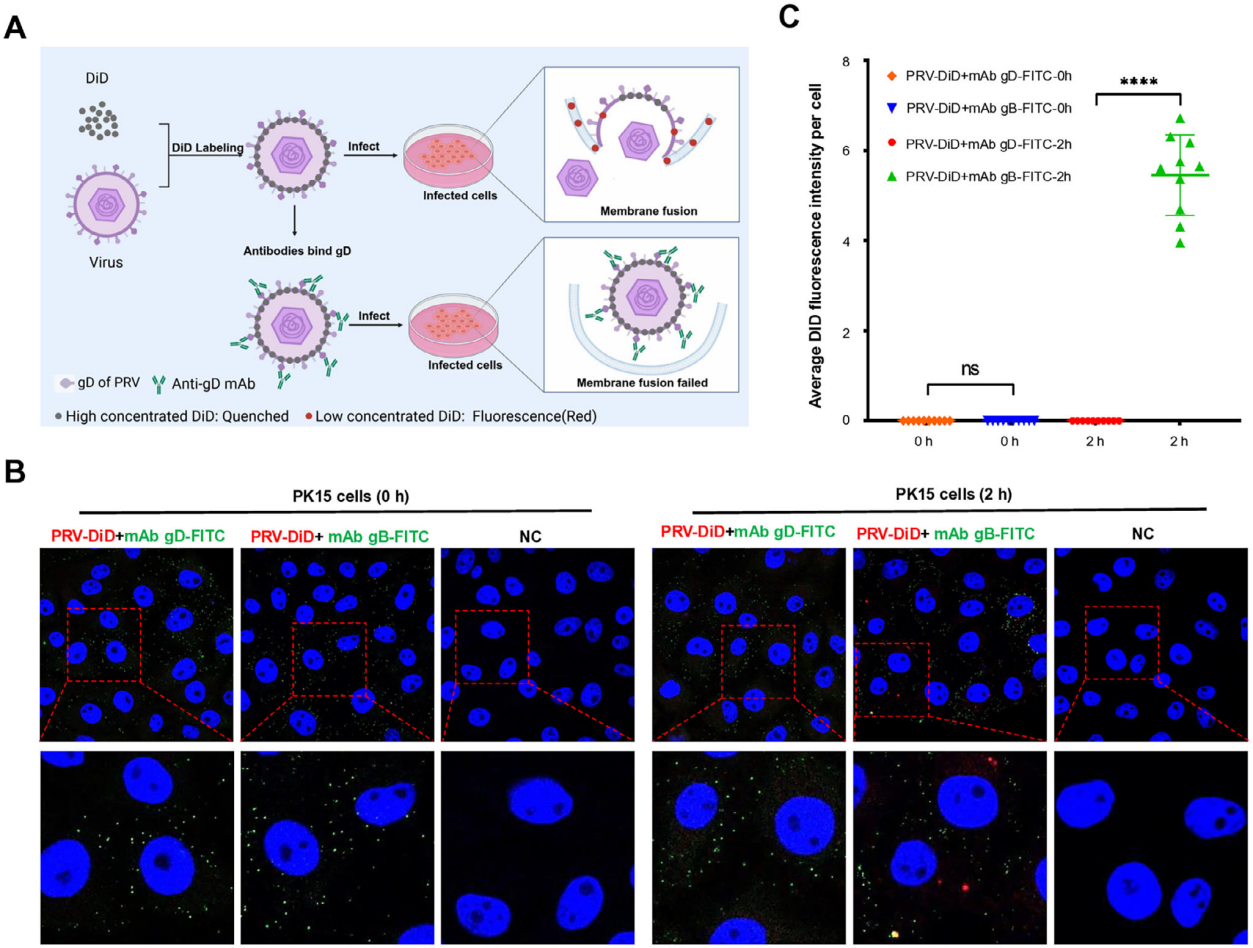
mAb 6F7 blocks viral membrane fusion. (A) Schematic diagram of the membrane fusion detection between the viral envelope and the cell membrane. High concentrations of DiD dye exhibit self‐quenching; when the viral envelope fuses with the cell membrane, the DiD concentration decreases, leading to the emission of red fluorescence. (B) Tracking viral membrane fusion via DiD dye release. PRV particles pre‐labeled with the high‐concentration lipophilic dye DiD were pre‐incubated with FITC‐conjugated mAb 6F7 (gD‐specific; 50 µg) or a FITC‐conjugated control mAb (gB‐specific; 50 µg) for 2 h at 37°C. The mixtures were added to pre‐chilled PK15 cells and incubated at 4°C for 2 h for synchronization. Cells were washed, fresh medium was added, and then shifted to 37°C. At indicated time points, cells were fixed, stained with DAPI (blue), and visualized by confocal microscopy. FITC signal (green) indicates antibody localization, while DiD signal (red) indicates viral membrane fusion. Increase of DiD signal correlates with viral membrane fusion. The experiment was repeated three times, and a representative image is shown. (C) Quantification of viral membrane fusion. The average DiD fluorescence intensity per cell from ten random fields was calculated using ImageJ software. Data are presented as mean ± SD.

After viral internalization, the fusion of the viral envelope with the cell membrane occurs. To determine whether 6F7 exerts its neutralizing activity at the membrane‑fusion step, we employed a DiD‑based fusion assay. Purified PRV particles were labeled with the lipophilic dye DiD at a high concentration, which results in self‑quenching of fluorescence [22]. Upon fusion of the viral envelope with the cellular plasma membrane, the dye becomes diluted, relieving quenching and producing a detectable red signal (Figure 5A). PRV particles pre‑labeled with DiD were incubated for 2 h at 37°C with either FITC‑conjugated 6F7 against gD (50 µg; Figure S5) or a FITC‑conjugated control IgG directed against gB (50 µg). The antibody–virus mixtures were then added to pre‑chilled PK15 cells and allowed to bind for 2 h at 4°C. After washing, fresh medium was added, and the cells were shifted to 37°C to initiate entry. At defined time points, cells were fixed and examined by confocal microscopy. The green FITC signal marks the location of the bound antibody, whereas the red DiD signal reports viral‑envelope fusion; an increase in DiD fluorescence corresponds to successful membrane fusion.

Confocal images revealed that cells treated with 6F7 displayed a complete absence of DiD fluorescence, indicating that viral‑envelope–cell‑membrane fusion was fully blocked (Figure 5B). Quantitative analysis of DiD intensity (average fluorescence per cell across ten random fields) using ImageJ confirmed a statistically significant reduction in fusion events in the 6F7 group compared with the control IgG group (Figure 5C). These findings demonstrate that 6F7 neutralizes PRV by blocking viral‑envelope fusion with the host cell membrane, rather than interference with attachment or internalization.

### 2.6 6F7 also Inhibits Virus Cell‑to‑Cell Spread

**FIGURE 6:**
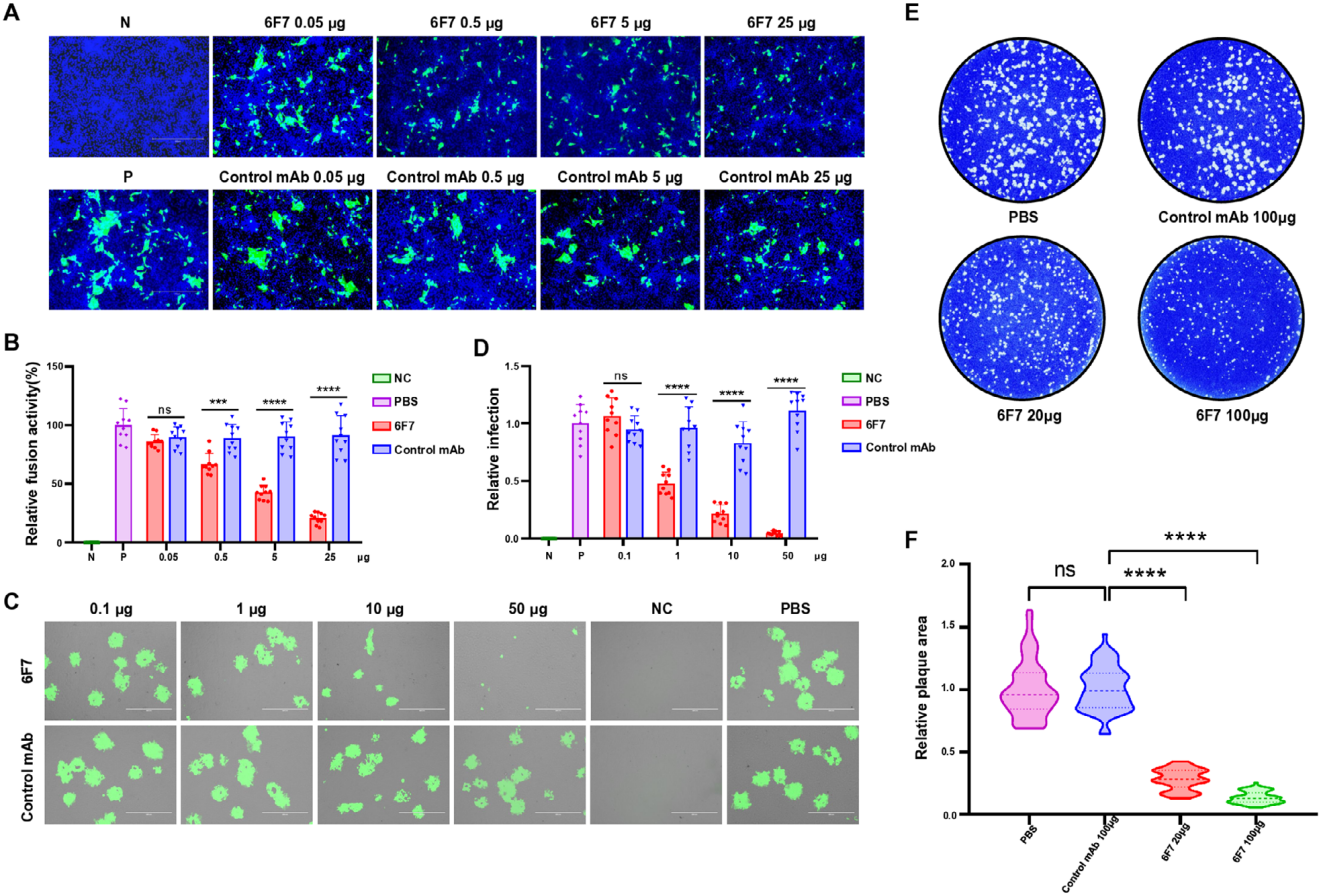
mAb 6F7 inhibits glycoprotein‐mediated and PRV‐induced cell‐to‐cell fusion. (A) Inhibition of virus‐free cell fusion. RK13 cells in 24‐well plates were co‐transfected with plasmids expressing EGFP (20 ng), PRV glycoproteins gB, gD, gH, and gL (100 ng each), and cultured in medium containing mAb 6F7 or a control antibody (0.05 µg, 0.5 µg, 5 µg, or 25 µg). After 36 h, cells were stained with DAPI and visualized by fluorescence microscopy. The experiment was repeated three times, and a representative image is shown. Scale bars, 400 µm. (B) The relative fusion area, based on EGFP‐positive syncytia, was quantified using ImageJ software. The fusion area in the PBS control was set to 100%. (C) Inhibition of PRV spread via direct cell‐to‐cell transmission. Vero‐E6 cells in 12‐well plates were infected with PRV‐EGFP (MOI = 0.05) for 2 h at 37°C. After virus removal and washing, fresh medium containing mAb 6F7 or control antibody (0.1, 1, 10, or 50 µg) was added. PBS served as a control. At 24 h post‐infection, viral spread was visualized by fluorescence microscopy. The experiment was repeated three times, and a representative image is shown. Scale bars, 400 µm. (D) The relative EGFP area was quantified. The EGFP area in the PBS control was set to 1.0. (E) Inhibition of plaque formation. PK15 cells in 6‐well plates were infected with PRV HeN1 (MOI = 0.005) for 2 h. Cells were then overlaid with medium containing FBS, low‐melting‐point agarose, and mAb 6F7 (20, 100 µg) or control mAb (100 µg). After 72 h, plaques were visualized by crystal violet staining. The experiment was repeated three times, and a representative image is shown. (F) The relative plaque area was quantified using ImageJ. The plaque area in the PBS control was set to 1.0.

Because 6F7 blocks viral‑envelope–cell‑membrane fusion, we hypothesized that it might also impede the cell‑to‑cell transmission pathway, which is a critical component of PRV dissemination. First, we confirmed that gD is indispensable for mediating membrane fusion between infected and neighboring cells; in the absence of gD, the fusogenic activities of gB and the gH/gL complex are markedly reduced (Figure S6). To assess the effect of 6F7 on cell‑cell fusion, we established a quantitative membrane‑fusion assay using RK13 cells [23, 24, 25]. Cells in a 24‐well plate were transfected with plasmids encoding gD, gB, and gH/gL to induce syncytium formation, and then incubated with increasing concentrations of 6F7 or a control mAb. Compared with the control antibody, 6F7 caused a dose‑dependent decline in the fusogenic capacity of the gD‑gB‑gH/gL combination (Figure 6A,B).

We next examined the impact of 6F7 on the cell‑to‑cell spread of infectious virus. PRV‑EGFP (MOI  =  0.05) was allowed to infect Vero‐E6 cells in a 12‐well plate for 2 h at 37°C; after removal of inoculum and washing, fresh medium containing 6F7 or control antibody at 0.1 µg, 1 µg, 10 µg, or 50 µg (PBS as a control) was added. At 24 h post‑infection, viral dissemination was visualized by fluorescence microscopy. While the number of plaques was unchanged, the average plaque size was significantly reduced in the presence of 6F7 (Figure 6C and 6D). To corroborate these observations with the wild‑type virus, PK15 cells in a 6‐well plate were infected with PRV HeN1 (MOI  =  0.005) for 2 h, then overlaid with medium containing 20 µg or 100 µg of 6F7, or 100 µg of control mAb, supplemented with fetal bovine serum and low‑melting‑point agarose. After 72 h, plaques were stained with crystal violet. Consistent with the EGFP assay, 6F7 markedly reduced plaque size without affecting plaque number (Figure 6E,F). Collectively, these data demonstrate that 6F7 not only blocks viral‑envelope fusion with the host cell membrane but also impairs PRV cell‑to‑cell spread, highlighting its potential as a broad‑spectrum antiviral agent.

### 2.7 6F7 Interferes with the Interaction Between gD and the Cellular Receptor Nectin‑1

**FIGURE 7:**
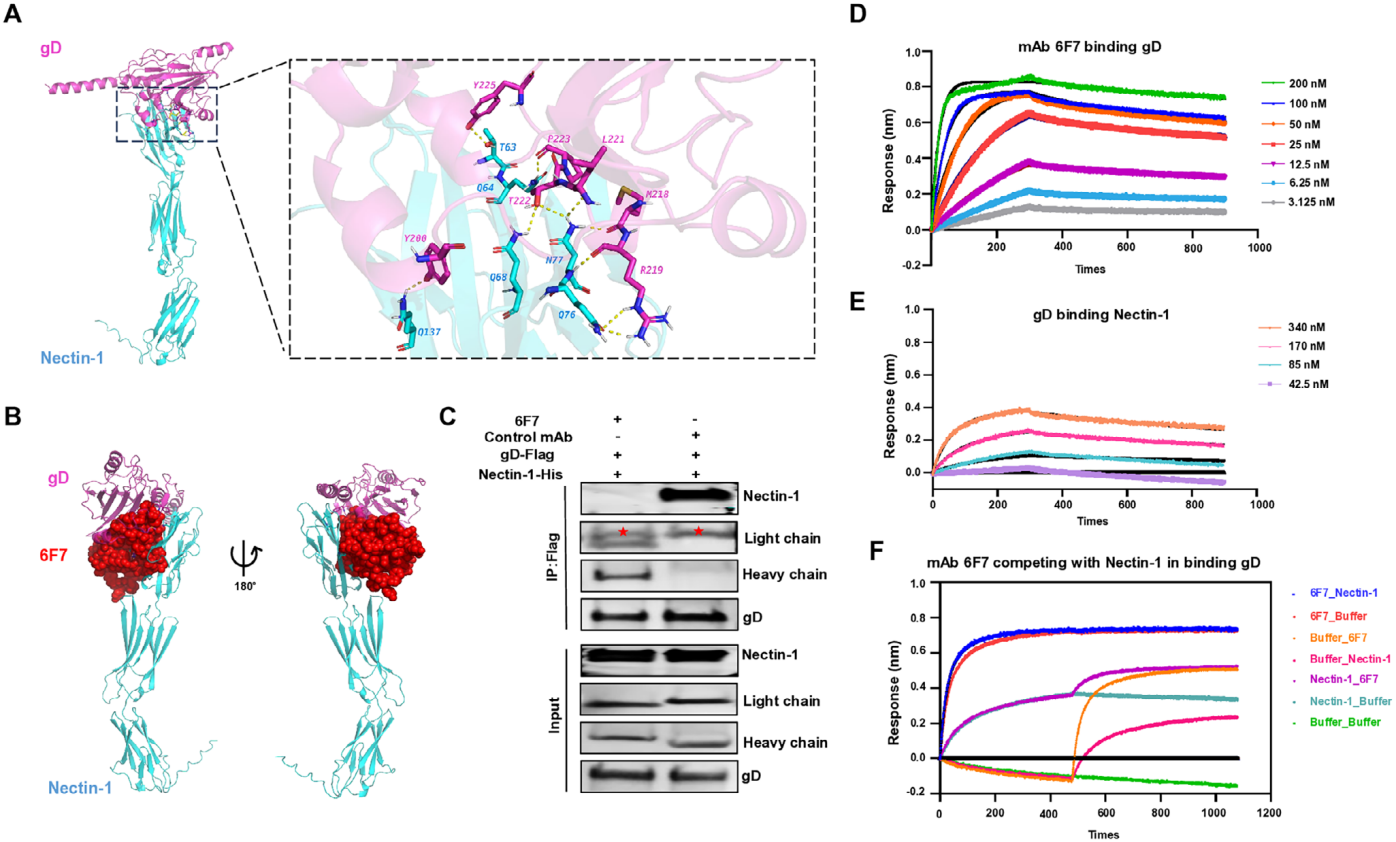
The mAb 6F7 blocked gD binding to the receptor Nectin‐1. (A) Cartoon representation of the 3D structure of PRV gD (pink) in complex with Nectin‐1 (light blue). (B) Structural model demonstrating steric hindrance. The model shows that binding of mAb 6F7 Fab (red, surface representation) to gD (pink, cartoon representation) occludes the interface required for Nectin‐1 (light blue, cartoon representation) engagement. (C) Co‐immunoprecipitation (Co‐IP) assay confirming blockade of gD‐Nectin‐1 interaction. Purified gD‐Flag (20 µg), Nectin‐1‐His (20 µg), and mAb 6F7 (50 µg) or control mAb (50 µg) were incubated together. Protein complexes were immunoprecipitated using anti‐Flag Fab agarose beads and analyzed by western blotting. The band labeled with an asterisk represents the Light chain of the immunoprecipitating Flag Fab antibody. The experiment was repeated three times, and a representative image is shown. (D) Binding affinity of gD for mAb 6F7 using bio‐layer interferometry (BLI) on an Octet platform. The mAb 6F7 was loaded onto the sensor surface, and association was measured by exposing the sensor to serial dilutions of recombinant gD. (E) Binding affinity of gD and its cellular receptor, Nectin‐1, using anti‐His biosensors (GatorBio). The His‐tagged gD was captured, and association and dissociation with varying concentrations of Nectin‐1 were recorded. (F) The competition between mAb 6F7 and Nectin‐1 for gD binding. Anti‐His biosensors were immobilized with recombinant PRV gD‐His, then the sensors were first saturated with mAb 6F7 or Nectin‐1, followed by exposure to the second ligand (or buffer control. All sensorgram data were processed and fitted using the Gator data analysis suite (GatorBio). The experiment was repeated three times, and a representative image is shown.

To further elucidate the neutralizing mechanism of mAb 6F7, we investigated its potential to block the interaction between PRV gD and its cellular receptor Nectin‑1. Using AlphaFold 3, we first generated a structural model of the PRV gD–Nectin‐1 complex, in which gD is shown in pink and Nectin‑1 in light blue (Figure 7A). The resulting AlphaFold 3 model was in excellent agreement with the previously validated crystal structure (Figure S7) [14]. Docking of the Fab fragment of 6F7 into this model revealed significant steric clashes, indicating that antibody binding would physically occlude the receptor‐binding site on gD and prevent Nectin‑1 engagement (Figure 7B).

We next validated this computational prediction experimentally using co‑immunoprecipitation (Co‑IP). Purified gD‑Flag and Nectin‑1‑His were incubated with either mAb 6F7 or an isotype control antibody (50 µg each). Complexes were pulled down with anti‑Flag Fab agarose beads and analyzed by Western blotting. In control samples, a strong Nectin‑1‑His signal co‑precipitated with gD‑Flag, confirming the specific interaction. In contrast, samples treated with 6F7 showed markedly reduced co‑precipitation of Nectin‑1, demonstrating that 6F7 effectively disrupts the gD–Nectin‐1 complex (Figure 7C).

To quantitatively assess the binding characteristics, we performed bio‐layer interferometry (BLI). The affinity of 6F7 for immobilized gD was measured, yielding a dissociation constant (*K_D_*) of 9.2 nM, indicative of high‐affinity binding (Figure 7D). For competitive binding assays, we established baseline binding of gD to soluble Nectin‐1 (Figure 7E). We observed that 6F7 still bound efficiently to gD pre‐saturated with Nectin‐1, albeit with a moderately decreased association rate, confirming that 6F7 competes with the receptor for gD binding (Figure 7F). Collectively, these data consistently demonstrate that mAb 6F7 sterically hinders the gD–Nectin‐1 interface, thereby blocking receptor engagement and contributing to its potent neutralizing activity.

### 2.8 Humanized 6F7 Demonstrates Potent Therapeutic Efficacy against PRV Infection

**FIGURE 8:**
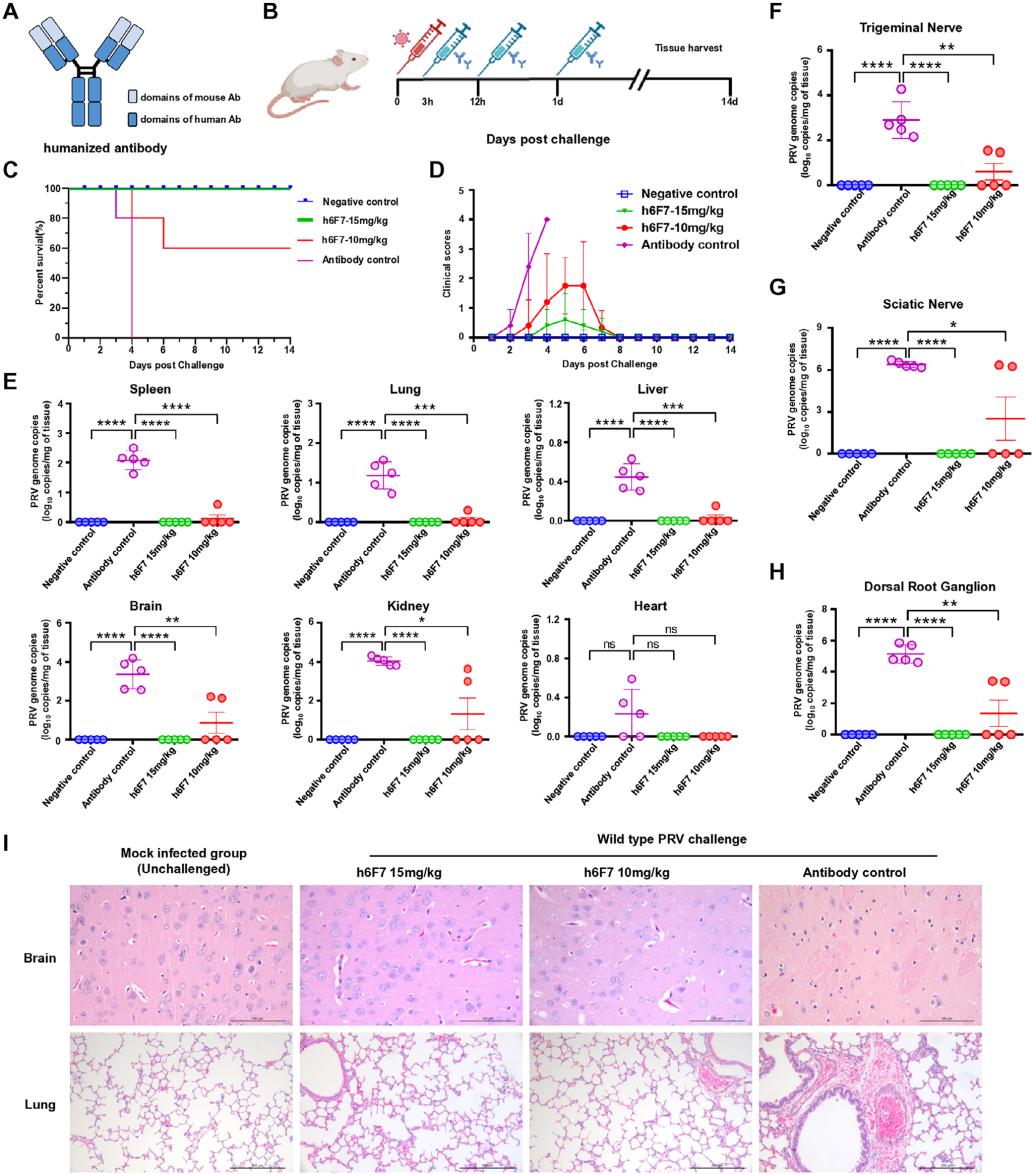
Therapeutic efficacy of a humanized 6F7 antibody in a murine model of PRV infection. (A) Schematic of the humanized 6F7 antibody construct. Mouse‐derived variable domains are shown in light blue; human constant domains are in blue. (B) Experimental timeline for PRV challenge and antibody treatment in mice. (C) Survival curves of mice infected with PRV and subsequently treated with humanized 6F7 or a control antibody. (D) Clinical scores of infected mice following treatment with humanized 6F7 or control antibody. (E) Viral load quantification by qRT‐PCR in various tissues of treated mice: systemic organs (spleen, lung, liver, brain, kidney, heart), (F) trigeminal nerve, (G) dorsal root ganglion, and (H) sciatic nerve. The above data are replicates of 5 mice, and the data are presented as Mean ± SD. (I) Histopathological analysis of five mice/group and representative hematoxylin and eosin (H&E)‐stained sections of brain and lung tissues from PRV‐infected mice treated with humanized 6F7 or control antibody was shown. Scale bars of the brain, 100 µm; scale bars of the lung, 200 µm.

To facilitate clinical translation, the murine monoclonal antibody 6F7 was humanized as depicted in Figure 8A. The therapeutic efficacy of the humanized antibody was subsequently assessed in a mouse model of PRV infection. Four experimental groups were established: (i) a negative control (uninfected), (ii) an isotype control (infected, treated with non‑specific IgG), and two treatment groups receiving the humanized 6F7 antibody at doses of 10 mg/kg or 15 mg/kg, respectively (Figure 8B). Mice administered humanized 6F7 at 10 mg/kg exhibited a survival rate of 60 %, whereas all animals in the isotype‑control group succumbed to infection (Figure 8C). The 15 mg/kg cohort achieved complete (100 %) survival, indicating a dose‑dependent protective effect. Throughout the observation period, all surviving mice in both treatment groups displayed mild clinical signs, in stark contrast to the high clinical scores recorded for the control group (Figure 8D). The qRT‑PCR analysis demonstrated a significant reduction in viral DNA load across systemic organs—including the spleen, lung, liver, brain, kidney, and heart—of treated animals compared with controls (Figure 8E). Moreover, viral DNA was undetectable in three distinct peripheral ganglia (trigeminal nerve, dorsal root ganglion, and sciatic nerve), indicating that the antibody completely prevented latent infection in neuronal tissues (Figure 8F‑‐H). Histopathological examination via hematoxylin‑eosin staining revealed severe inflammatory infiltrates, necrosis, and tissue disruption in brain and lung sections from control mice. In contrast, tissues from both h6F7‑treated groups, particularly the 15 mg/kg cohort, showed largely preserved tissue architecture with minimal inflammatory changes (Figure 8I). Taken together, these results support the continued development of humanized 6F7 as a promising therapeutic antibody against PRV infection.

## 3 Discussion

The emergence of PRV as a zoonotic pathogen has reshaped the traditional view of this virus, which was historically regarded as a veterinary‐restricted agent [2, 3, 26, 27, 28]. While vaccination represents a cornerstone strategy for controlling PRV circulation in domestic pigs and thereby substantially mitigates the risk of zoonotic transmission to humans, no effective therapeutic interventions are currently available for the treatment of established PRV infection in humans [29, 30, 31]. Here, we report the development of a humanized, broadly neutralizing monoclonal antibody, h6F7, that targets the PRV gD and confers complete post‑exposure protection in a lethal murine model. Accumulating evidence has established that both gD and gB serve as critical targets for eliciting potent and broad neutralizing antibodies against alphaherpesvirus [14, 15, 17, 32]. Our work further validates gD as a highly effective target for antibody‑mediated neutralization. Notably, a recent study by Wang et al. identified a broadly neutralizing antibody targeting the conserved Domain I of alphaherpesvirus gB that confers cross‐genus protection against multiple members of the *Alphaherpesvirinae* subfamily, including HSV‐1, HSV‐2, and PRV [17]. Inspired by this work, we further evaluated the cross‐reactivity of antibody 6F7 against HSV‐1 using an in vitro neutralization assay. Our results showed that 6F7 exhibited no detectable neutralizing activity against HSV‐1, indicating that its neutralizing capacity is highly specific to PRV (Figure S8). Although HSV‐1, HSV‐2, and VZV are closely related to PRV, the epitope in gD recognized by 6F7 appears to be less conserved across these viral species. Therefore, unlike the broadly reactive antibody described by Wang et al., 6F7 represents a PRV‐specific neutralizing antibody rather than a broad‐spectrum anti‐alphaherpesvirus agent [17].

The neutralizing activity of 6F7 (IC_50_ = 2.966‐5.558 nM) surpasses or is comparable to the most potent anti‑PRV antibodies reported to date. Importantly, 6F7 neutralizes PRV efficiently in the absence of complement, highlighting its intrinsic antiviral function. Epitope mapping revealed that 6F7 recognizes a conserved linear epitope and the key residues centered on P223, F224, Y225, and Q226 in gD, rationalizing its broad‑spectrum activity across all major PRV genotypes. In the future, resolving the high‐resolution crystal structure of the 6F7‐gD complex would provide unprecedented mechanistic details. Furthermore, the linear epitope targeted by h6F7 holds substantial promise for the development of epitope‐based vaccines against PRV, laying a solid foundation for the design of next‐generation prophylactic interventions with high specificity and immunogenicity.

A key mechanistic advantage of 6F7 lies in its ability to block viral‑envelope–cell‑membrane fusion without interfering with viral attachment or internalization. Herpesviruses, including PRV, exploit cell‑to‑cell spread as a major dissemination route, which can evade antibodies that only neutralize free virions [33, 34]. By directly inhibiting the fusion machinery, 6F7 effectively suppresses both cell‑free infection and cell‑to‑cell transmission, as demonstrated by the marked reduction in plaque size and syncytium formation.

In vivo, h6F7 conferred robust, dose‑dependent protection in a post‑exposure therapeutic model, with 100% survival achieved at a dose of 15 mg/kg. Of note, humanization of 6F7 endows this antibody with pivotal translational merits for prospective clinical applications in humans, as it drastically reduces the risk of adverse immunogenicity elicited by non‑human monoclonal antibodies, thereby permitting safe and repeated administration when clinically necessary. Moreover, the human IgG Fc domain within h6F7 enables engagement with the neonatal Fc receptor (FcRn), a well‑established lifelong regulator of IgG that extends serum half‑life via pH‑dependent cellular recycling mechanisms [35, 36, 37, 38]. This FcRn‑mediated recycling pathway is expected to prolong systemic antibody exposure and sustain durable therapeutic activity in vivo, further augmenting its clinical efficacy against PRV infection. The marked reduction in tissue viral loads likely curtails unrestrained viral replication, thereby alleviating downstream pathological damage, including neuronal death and cerebral edema. Latent infection is an important characteristic of PRV, and once latency is established, it is difficult to eliminate [39]. Of profound therapeutic significance, h6F7 effectively prevented the establishment of latent infection within the examined nervous system tissues. This hallmark finding represents a distinct therapeutic edge over conventional antiviral agents.

In the present study, we evaluated post‐exposure protection when the antibody was administered 3 h after PRV challenge. While longer treatment intervals beyond this time point have not yet been investigated, our findings support the notion that earlier antibody intervention correlates with improved therapeutic efficacy against lethal PRV infection. Although our studies were conducted in mice, several features support the translational potential of h6F7. The antibody is humanized to minimize immunogenicity, exhibits high affinity for PRV gD, and mechanistically disrupts the gD‐Nectin‑1 interaction—a receptor pathway conserved in humans. Moreover, the strategy of targeting a conserved fusion‑associated epitope may be generalizable to other neurotropic herpesviruses, such as HSV and VZV, where analogous entry mechanisms operate.

Despite the promising efficacy of 6F7 demonstrated in our study, a notable limitation is the absence of direct comparison with other reported therapeutic anti‐PRV antibodies in the post‐exposure therapeutic model and cell‐to‐cell spread assay. This constraint arises from the lack of commercially available neutralizing or therapeutic antibodies against PRV, as well as the limited accessibility of previously described antibodies, which are often non‐humanized or not shared as research reagents. Humanized anti‐PRV antibodies, in particular, remain scarce and unavailable for external validation. To contextualize our findings, we compared the neutralization potency of 6F7 with published data: Wang et al. reported a gB‐targeting antibody with an IC_50_ of 19.77 µg/mL [17], Li et al. described gB‐specific antibodies with IC_50_ values ranging from 15.2 µg/mL to 31.6 µg/mL [14], and Zhang et al. identified a gD‐targeting antibody with an IC_50_ of 2.514 µg/mL [19]. In contrast, 6F7 exhibited a significantly lower IC_50_ of 2.966 nm (0.4402 µg/mL), indicating superior neutralizing activity. These comparative analyses, while indirect, strongly support 6F7 as a highly potent candidate for anti‐PRV intervention.

In summary, we have developed a monoclonal antibody, 6F7, that potently neutralizes PRV by blocking membrane fusion and cell‑to‑cell spread. Its ability to reduce viral loads and prevent latent infection in a post‑exposure setting positions h6F7 as a promising clinical candidate for human PRV infection. These findings also provide a strategic framework for the design of antibody‑based therapeutics against neurotropic herpesviruses.

## 4 Materials and Methods

### 4.1 Ethics Statement

All animal experiments received approval from the Committee on the Ethics of Animal Experiments at the Harbin Veterinary Research Institute, Chinese Academy of Agricultural Sciences (CAAS; Approval No. 250526‐03‐GR).

### 4.2 Cell, Viruses, Protein, and Antibody

African green monkey kidney cells (Vero‐E6, ATCC CRL‐1586), human embryonic kidney cells (HEK293T, ATCC CRL‐11268), rabbit kidney cells (RK13, ATCC CCL‐37), porcine kidney‐15 cells (PK15, ATCC CCL‐33), and human cervical carcinoma cells (HeLa, ATCC CCL‐2) were maintained in Dulbecco's modified Eagle's medium (DMEM; Gibco, USA) supplemented with 10% fetal bovine serum (FBS; Gibco, USA) at 37°C in a 5% CO_2_ atmosphere.

The PRV strains HeN1 (GenBank KP098534) and SC (GenBank KT809429) were preserved in our lab. TJ strain (GenBank KJ789182) was generously provided by Professor Yuan Sun of our institute. The Bartha K61 strain (GenBank JF797217) and a PRV HeN1 strain expressing enhanced green fluorescent protein (EGFP) were preserved at −80°C; the latter was maintained as previously reported [15, 40].

For protein expression, gD‐Flag and Nectin‐1‐Flag‐hFc were produced in HEK293F cells and purified via Flag tags. Similarly, gD‐His and Nectin‐1‐His were expressed in HEK293F cells and purified using a His tag.

An antibody targeting PRRSV nsp2 served as an isotype control [41]. Antibody purification was performed using Protein A/G magnetic beads (MCE, USA) according to the manufacturer's protocol. The humanized version, h6F7, was expressed in CHO cells and purified by Biointron (Taizhou, China).

### 4.3 Mouse Immunization and Monoclonal Antibody Screening

BALB/c mice were immunized with gD‑encoding mRNA vaccine encapsulated in lipid nanoparticles (LNPs) at a dose of 10 µg per mouse for three times at 14‐day intervals [15]. One week after the third immunization, blood samples were collected, and serum antibody titers were determined by indirect ELISA. In brief, gD protein (100 ng/well) was coated onto 96‐well ELISA plates and incubated overnight at 4°C. After washing with PBST, the plates were blocked with PBS containing 5% nonfat milk (200 µL/well) at 37°C for 2 h. Serially diluted mouse serum samples (1:1000 to 1:64,000; 100 µL/well) were added and incubated at 37°C for 1 h. Subsequently, HRP‐conjugated goat anti‐mouse IgG secondary antibody (1:10,000; Zhongshan Golden Bridge, China) was added (100 µL/well) and incubated at 37°C for 45 min. After three final PBST washes, TMB substrate solution was added (100 µL/well) and incubated for 10 min at 37°C in the dark. The reaction was terminated with 0.5 m HCl (50 µL/well), and the absorbance at 450 nm was measured within 10 min to determine serum titers.

Mice exhibiting high serum titers were selected, and splenocytes were harvested for fusion with SP2/0 myeloma cells 3 days after booster immunization. The fused cells were cultured in DMEM medium supplemented with 20% fetal bovine serum (FBS) and 1% hypoxanthine‐aminopterin‐thymidine (HAT, Sigma–Aldrich, USA). After 7–10 days of culture, positive clones reactive with PRV gD protein were screened by ELISA as described above. Then, viral inhibition assays were performed using the supernatant of hybridoma cells, and clone 6F7 showed the strongest inhibitory activity against the virus and used for subsequent studies. In addition, the reactivity of monoclonal antibodies with other PRV strains (TJ, SC, and Bartha K61) was evaluated by indirect IFA. In brief, Vero‐E6 cells were seeded in 12‐well or 96‐well plates. When cell confluence reached 90%–100%, cells were infected with PRV strains (HeN1, TJ, SC, or Bartha K61) at a multiplicity of infection (MOI) of 0.01. Upon observation of obvious cytopathic effect (CPE), cells were fixed, permeabilized, and blocked. Cells were incubated with mAb supernatant, or SP2/0 supernatant (negative control), followed by an FITC‐conjugated goat anti‐mouse IgG secondary antibody (1:200; Sigma, USA).

### 4.4 Neutralization Assay with or Without Complement

The neutralization capacity of mAbs against various PRV strains was evaluated as described previously [15]. Briefly, Vero‐E6 cells were seeded in 96‐well plates. Serial dilutions of mAb 6F7 or the control antibody in DMEM were prepared and mixed with an equal volume of virus suspension (200 TCID_50_ of HeN1, SC, TJ, or Bartha K61 strain), followed by incubation at 37°C for 2 h. Then, 100 µL of each mixture was transferred to the cell monolayers. After a 2 h adsorption period at 37°C, the inoculum was removed, cells were washed with PBS, and fresh medium was added. Plates were incubated for 48 h at 37°C with 5% CO_2_. To determine whether the neutralization activity was complement‐dependent, 5% fresh non‐immune mouse serum (56°C heat‐inactivated or not) was added into the virus‐antibody mixture [19]. The 50% inhibitory concentration (IC_50_) was determined using GraphPad Prism 8.0.

### 4.5 FITC Labeling of mAbs

Conjugation of mAbs gD (6F7) and gB with fluorescein isothiocyanate (FITC) was performed using a commercial kit (Sangon Biotech, China) per the manufacturer's instructions. Purified antibodies were incubated with FITC solution at 37°C for 90 min in the dark, followed by purification through a desalting column. Successful labeling was confirmed by IFA.

### 4.6 Attachment Assays

The ability of mAb 6F7 to inhibit PRV attachment was assessed with modifications to established protocols [19, 42, 43]. HeLa, PK15, Vero‐E6, or HEK293T cells were seeded in 12‐well plates. Upon reaching 80–90% confluence, cells were washed with cold PBS and pre‐chilled at 4°C for 15 min. PRV HeN1 (at MOIs of 0.01, 0.1, or 1) was pre‐incubated with mAb 6F7 or control mAb (0.1 µg, 1 µg, or 10 µg) for 2 h at 37°C. The mixtures were then added to the chilled cells and incubated at 4°C for 2 h to allow attachment. The unbound virus was removed by washing with cold PBS. Furthermore, an additional control experiment using citric acid (pH 2–3) to remove unbound virus. Cell‐associated viral DNA levels were quantified by qRT‐PCR, and bound virions were visualized by confocal microscopy.

### 4.7 Internalization Assays

Cells were prepared for the attachment assay. After the 2 h virus‐antibody adsorption period at 4°C, cells were washed with cold PBS or citric acid, replenished with fresh pre‐warmed medium, and shifted to 37°C for 2 h to permit internalization. Subsequently, cells were washed with cold PBS or citric acid, and the internalized virus was quantified by qRT‐PCR and visualized by confocal microscopy.

### 4.8 DiD Labeling and Viral Membrane Fusion Assay

Vero‐E6 cells at full confluence in DMEM with 2% FBS were infected with PRV HeN1 (MOI = 0.01). Upon extensive CPE appearance, the supernatant was harvested, clarified by centrifugation (6,000 × *g*, 20 min, 4°C), and subjected to ultracentrifugation (25 000 rpm, 3 h, 4°C; Type 45Ti rotor). The pellet was resuspended in dye buffer and labeled with DiD (Beyotime, China) for 2 h at room temperature in the dark with gentle agitation [22]. Free dye was removed by ultracentrifugation (30 000 rpm, 16–18 h, 4°C; SW41Ti rotor) through a 50% (w/v) CsCl gradient. The visible virus band was collected, concentrated further (25 000 rpm, 3 h, 4°C; SW32Ti rotor), filtered (0.45 µm), and stored at −80°C.

To assess the effect of mAb on viral membrane fusion, PRV‐DiD (10 MOI) was incubated with 50 µg of mAb gD‐FITC (6F7) or control mAb gB‐FITC for 2 h at 37°C. The mixtures were added to pre‐chilled PK15 cells for 2 h at 4°C. After washing, fresh medium was added, and cells were incubated at 37°C for 0 or 2 h. Cells were then fixed, nuclei were stained with DAPI, and viral membrane fusion was analyzed by confocal microscopy. DiD fluorescence intensity per cell was quantified using ImageJ.

### 4.9 Inhibition of Virus‐Free Cell‐Cell Fusion

RK13 cells at 80–90% confluence in 24‐well plates were co‐transfected with 20 ng pDC315‐EGFP and 100 ng each of pCAGGS‐HA‐gB, ‐gD, ‐gH, and ‐gL, as described [23, 24, 25, 44, 45]. Six hours post‐transfection, the medium was replaced with fresh medium containing mAb 6F7 or control antibody (0.05 µg, 0.5 µg, 5 µg, or 25 µg). After 36 h, syncytia formation was observed by fluorescence microscopy. The relative fusion area, based on EGFP signal, was quantified with ImageJ, with the PBS control set as 100%.

### 4.10 Inhibition of PRV‐Mediated Cell–Cell Fusion

Vero‐E6 cells in 12‐well plates were infected with PRV‐EGFP (MOI = 0.05). After 2 h at 37°C, the inoculum was removed, cells were washed, and fresh medium containing mAb 6F7 or control antibody (0.1 µg, 1 µg, 10 µg, or 50 µg) or PBS was added. Viral spread was assessed by EGFP fluorescence 24 h post‐infection. The relative infection area was quantified (ImageJ), normalized to the PBS control (set as 1.0).

For plaque reduction, PK15 cells in 6‐well plates were infected with PRV HeN1 (MOI = 0.005). After 2 h at 37°C, the inoculum was removed, cells were washed and overlaid with DMEM containing FBS, low‐melting‐point agarose, and mAb 6F7 (20 µg or 100 µg) or control mAb (100 µg). After 72 h at 37°C (inverted culture), plaques were stained with crystal violet, and their relative areas were measured (ImageJ), with the PBS control set as 1.0.

### 4.11 Identification of mAb 6F7 Epitope

Site‐directed mutagenesis was performed on the amino acid residues of gD that may be involved in mAb binding (based on structural models), generating mutants gD‐Y35A, ‐E37A, ‐W39A, ‐T136A, ‐D140A, ‐Q196A, ‐R219A, ‐F220A, ‐L221A, ‐T222A, ‐P223A, ‐F224A, ‐Y225A, ‐Q226A, ‐Q227A, and ‐P228A, alongside wild‐type (WT) gD, all cloned into pB513B with a C‐terminal Flag tag. HeLa cells transfected with these constructs for 24 h were fixed (4% PFA, 30 min, RT), permeabilized (0.25% Triton X‐100, 15 min, RT), and blocked (2% BSA in PBS, 1 h, 37°C). Cells were incubated with 10 µg/mL mAb 6F7 or anti‐Flag antibody (Proteintech, USA) for 1 h at 37°C, followed by FITC‐conjugated goat anti‐mouse IgG (1:200; Sigma, USA) for 1 h at 37°C. Nuclei were counterstained with DAPI, and fluorescence was visualized by microscopy. HEK293T cells transfected with these constructs for 24 h were lysed in cell lysis buffer (50 mm Tris‐HCl, 150 mm NaCl, 1 mm EDTA, 1% Triton X‐100, pH 7.4) for 30 min at 4°C, followed by western blotting analysis, with mAb 6F7 or anti‐Flag antibody used as the primary antibody.

### 4.12 Co‐Immunoprecipitation (Co‐IP)

To determine if mAb 6F7 disrupts the gD–Nectin‐1 interaction, gD‐Flag (20 µg) and Nectin‐1‐His (20 µg) were incubated with mAb 6F7 or control mAb (50 µg each) for 1 h at 4°C. Complexes were immunoprecipitated using Flag Fab agarose beads (ChromoTek, Germany) for 2 h at 4°C, washed three times with wash buffer (10 mm Tris‐HCl, 150 mm NaCl, 0.05% Nonidet P‐40, 0.5 mm EDTA, pH 7.5), and analyzed by western blotting.

### 4.13 Western Blotting

The western blotting was performed as described in our previous reports [46, 47, 48]. Briefly, proteins were separated by SDS‐PAGE and transferred to PVDF membranes (Millipore, USA). Membranes were blocked with 5% non‐fat milk in PBS (1 h, RT), incubated with primary antibody overnight at 4°C, washed, and incubated with appropriate secondary antibody (1 h, RT). Signals were detected using an Odyssey CLx imaging system.

### 4.14 Binding Kinetics and Affinity Analysis by Bio‐Layer Interferometry

Binding kinetics and affinity measurements for the mAb 6F7 against PRV gD were performed using bio‐layer interferometry (BLI) on an Octet platform as follows. Protein A biosensors (Sartorius) were hydrated and baseline‐equilibrated for 60 s in assay buffer (PBS, pH 7.4, 0.02% Tween 20, 1 mg/mL BSA). Antibody was then loaded onto the sensor surface at 11 µg/mL to a response level of 1.6‐1.8 nm. After a second baseline step in buffer (60 s), association was measured by exposing the sensor to serial dilutions of recombinant gD protein for 300 s, followed by dissociation monitoring in buffer for 600 s.

A parallel assay was conducted to characterize the interaction between PRV gD and its cellular receptor, Nectin‐1. Anti‐His biosensors (GatorBio) were equilibrated in the same assay buffer, followed by capture of tagged gD‐His (11 µg/mL) to a similar response level (1.6–1.8 nm). After baseline stabilization, association and dissociation with varying concentrations of Nectin‐1 were recorded using the same time intervals (300 s association, 600 s dissociation).

To evaluate potential competition between mAb 6F7 and Nectin‐1 for gD binding, a sequential binding assay was designed. Anti‐His biosensors were immobilized with recombinant PRV gD‐His (11 µg/mL) after pre‐equilibration in PBS containing 0.02% Tween‐20. Sensors were first saturated with 10 µg/mL of either mAb 6F7 or Nectin‐1, followed by exposure to the second ligand (or buffer control) for 300 s. All sensorgram data were processed and fitted using the Gator data analysis suite (GatorBio).

### 4.15 Structural Modeling

The amino acid sequences of PRV gD, mAb 6F7 (from hybridoma sequencing), and Nectin‐1 were used for homology‐based 3D structure prediction via the AlphaFold 3 server [20]. Models were visualized and analyzed using PyMOL.

### 4.16 Therapeutic Efficacy of h6F7 in Mice

Twenty 8‐week‐old BALB/c mice were randomly divided into four groups (*n* = 5). Groups 1–3 were challenged intramuscularly in the hind leg with 100 µL of PRV HeN1 (2.5 × 10^3^ PFU). Group 4 served as an unchallenged control. At 3, 12, and 24 h post‐challenge, Groups 1, 2, and 3 received intraperitoneal injections of 15 mg/kg h6F7, 10 mg/kg h6F7, or 15 mg/kg control antibody, respectively. Mice were monitored daily for clinical signs. Survivors were euthanized by CO_2_ inhalation at 14 days post‐challenge. Nine tissues (brain, lung, heart, liver, spleen, kidney, trigeminal nerve, dorsal root ganglion, sciatic nerve) were collected for viral DNA quantification by qRT‐PCR. Brain and lung samples underwent histopathological examination (H&E staining).

### 4.17 Quantitative Real‐Time PCR (qRT‐PCR)

Viral DNA from cells and tissues was extracted using the TIANamp Genomic DNA Kit (Tiangen, China). The PRV gB gene was quantified using previously described primers and probe [15, 25, 49]: forward, 5′‐acggcacgggcgtgatc‐3′; reverse, 5′‐actcgcggtcctcgagca‐3′; TaqMan probe, FAM‐ctcgcgcgacctcatcgagccctgcac‐MGB.

### 4.18 Statistical Analyses

Data were analyzed using GraphPad Prism 8.0. Significance was determined by *t*‐tests, with *p* < 0.05 considered significant (^*^ *p* < 0.05, ^**^ *p* < 0.01, ^***^ *p* < 0.001, ^****^ *p* < 0.0001).

## Conflicts of Interest

The authors declare no conflict of interest.

## Supporting information




**Supporting File**: advs75771‐sup‐0001‐SuppMat.docx.

## Data Availability

The data that support the findings of this study are available from the corresponding author upon reasonable request.

## References

[1] T. C. Mettenleiter , B. G. Klupp , T. Müller , K.‐J. Yoon , J. P. Teifke , and B. Ehlers , “Herpesviruses,” Diseases of Swine (2025): 615–644.

[2] T. Y. Wang , C. Li , X. H. Cai , T. Shan , and Y. D. Tang , “Spillover of Pseudorabies Virus Variants to Humans: An Urgent Call for Pseudorabies Eradication in Domestic Pigs,” The Lancet Microbe (2026): 101351, 10.1016/j.lanmic.2026.101351. 41759550

[3] G. Wong , J. Lu , W. Zhang , and G. F. Gao , “Pseudorabies Virus: A Neglected Zoonotic Pathogen in Humans?,” Emerging Microbes & Infections 8, no. 1 (2019): 150–154, 10.1080/22221751.2018.1563459. 30866769 PMC6455137

[4] C. Ye , Q. Z. Zhang , Z. J. Tian , et al., “Genomic Characterization of Emergent Pseudorabies Virus in China Reveals Marked Sequence Divergence: Evidence for the Existence of Two Major Genotypes,” Virology 483 (2015): 32–43, 10.1016/j.virol.2015.04.013. 25965793

[5] J. W. Ai , S. S. Weng , Q. Cheng , et al., “Human Endophthalmitis Caused By Pseudorabies Virus Infection, China, 2017,” Emerging Infectious Diseases 24, no. 6 (2018): 1087–1090, 10.3201/eid2406.171612. 29774834 PMC6004832

[6] X. Han , C. Ji , S. Tian , et al., “Neutralizing Antibodies Against Chikungunya Virus and Structural Elucidation of Their Mechanism of Action,” Nature Communications 16, no. 1 (2025): 9682, 10.1038/s41467-025-64687-2. PMC12583524 41184282

[7] N. Y. Pang , A. S. Pang , V. T. Chow , and D. Y. Wang , “Understanding Neutralising Antibodies Against SARS‐CoV‐2 and Their Implications in Clinical Practice,” Military Medical Research 8, no. 1 (2021): 47, 10.1186/s40779-021-00342-3. 34465396 PMC8405719

[8] S. L. Oliver , Y. Xing , D. H. Chen , et al., “A glycoprotein B‐Neutralizing Antibody Structure at 2.8 Å Uncovers a Critical Domain for Herpesvirus Fusion Initiation,” Nature Communications 11, no. 1 (2020): 4141, 10.1038/s41467-020-17911-0. PMC7435202 32811830

[9] L. M. Kauvar , K. Liu , M. Park , et al., “A High‐Affinity Native Human Antibody Neutralizes Human Cytomegalovirus Infection of Diverse Cell Types,” Antimicrobial Agents and Chemotherapy 59, no. 3 (2015): 1558–1568, 10.1128/AAC.04295-14. 25534746 PMC4325823

[10] X. Zhang , J. Hong , L. Zhong , et al., “Protective Anti‐gB Neutralizing Antibodies Targeting Two Vulnerable Sites for EBV‐Cell Membrane Fusion,” Proceedings of the National Academy of Sciences 119, no. 32 (2022): 2202371119, 10.1073/pnas.2202371119. PMC9371650 35917353

[11] M. P. Daumer , B. Schneider , D. M. Giesen , et al., “Characterisation of the Epitope for a Herpes Simplex Virus Glycoprotein B‐Specific Monoclonal Antibody with High Protective Capacity,” Medical Microbiology and Immunology 200, no. 2 (2011): 85–97, 10.1007/s00430-010-0174-x. 20931340

[12] R. J. Geraghty , C. Krummenacher , G. H. Cohen , R. J. Eisenberg , and P. G. Spear , “Entry of Alphaherpesviruses Mediated by Poliovirus Receptor‐Related Protein 1 and Poliovirus Receptor,” Science 280, no. 5369 (1998): 1618–1620, 10.1126/science.280.5369.1618. 9616127

[13] R. S. Milne , S. A. Connolly , C. Krummenacher , R. J. Eisenberg , and G. H. Cohen , “Porcine HveC, a Member of the Highly Conserved HveC/Nectin 1 Family, Is a Functional Alphaherpesvirus Receptor,” Virology 281, no. 2 (2001): 315–328, 10.1006/viro.2000.0798. 11277703

[14] X. Li , F. Yang , X. Hu , et al., “Two Classes of Protective Antibodies Against Pseudorabies Virus Variant Glycoprotein B: Implications for Vaccine Design,” PLOS Pathogens 13, no. 12 (2017): 1006777, 10.1371/journal.ppat.1006777. PMC5754140 29261802

[15] Y. Sun , S. J. Xu , Y. Zhou , et al., “A Glycoprotein D‐Targeted Lipid Nanoparticle‐Encapsulated mRNA Vaccine Elicits Strong Protective Immunity Against Pseudorabies Virus,” Journal of Virology 99 (2025): 0147225, 10.1128/jvi.01472-25. PMC12645995 41196061

[16] L. Na , Y. Sun , W. Y. Qiu , et al., “Development of a Competitive ELISA for Detecting Antibodies Against Pseudorabies Virus Glycoprotein D,” Transboundary and Emerging Diseases 2025, no. 1 (2025): 1263531, 10.1155/tbed/1263531. 41282572 PMC12634162

[17] G. Wang , Y. Li , C. Wu , et al., “A Broadly Neutralizing Antibody Confers Cross‐Genus Protection Against Alphaherpesviruses by Inhibiting gB‐Mediated Membrane Fusion,” Nature Communications 16, no. 1 (2025): 11144, 10.1038/s41467-025-66099-8. PMC12708680 41402251

[18] Y. Chen , H. Wang , J. Zhou , et al., “The Co‐Application of Nectin‐1 and PRV gD Monoclonal Antibodies: An Effective Approach to Block Pseudorabies Virus Invasion,” Journal of Virological Methods 340 (2025): 115271, 10.1016/j.jviromet.2025.115271. 41086918

[19] T. Zhang , Y. Liu , Y. Chen , et al., “A Monoclonal Antibody Neutralizes Pesudorabies Virus by Blocking gD Binding to the Receptor Nectin‐1,” International Journal of Biological Macromolecules 188 (2021): 359–368, 10.1016/j.ijbiomac.2021.07.170. 34339791

[20] J. Abramson , J. Adler , J. Dunger , et al., “Accurate Structure Prediction of Biomolecular Interactions with AlphaFold 3,” Nature 630, no. 8016 (2024): 493–500, 10.1038/s41586-024-07487-w. 38718835 PMC11168924

[21] C. Chen , Y. Wu , J. Li , et al., “TBtools‐II: A “One for All, All for One” Bioinformatics Platform for Biological Big‐Data Mining,” Molecular Plant 16, no. 11 (2023): 1733–1742, 10.1016/j.molp.2023.09.010. 37740491

[22] Y. Z. Bai , H. Xu , Y. G. Liu , et al., “Nsp2 Replicase‐Mediated Viral Uncoating in Porcine Alveolar Macrophages Contributes to the Attenuation of PRRSV‐2 Live Attenuated Vaccine,” Journal of Virology 99 (2025): 0063625, 10.1128/jvi.00636-25. PMC12456151 40757848

[23] C. H. Rao , R. Huang , Y. Z. Bai , et al., “MARCH8 Inhibits Pseudorabies Virus Replication by Trapping the Viral Cell‐to‐Cell Fusion Complex in the Trans‐Golgi Network,” International Journal of Biological Macromolecules 274, no. Pt 2 (2024): 133463, 10.1016/j.ijbiomac.2024.133463. 38944094

[24] R. Huang , C. H. Rao , Y. Z. Bai , et al., “MARCH1 and MARCH2 Inhibit Pseudorabies Virus Replication by Trapping the Viral Cell‐to‐Cell Fusion Complex in Trans‐Golgi Network,” Veterinary Microbiology 295 (2024): 110164, 10.1016/j.vetmic.2024.110164. 38936155

[25] M. Chen , M. H. Wang , X. G. Shen , et al., “Neuropilin‐1 Facilitates Pseudorabies Virus Replication and Viral Glycoprotein B Promotes Its Degradation in a Furin‐Dependent Manner,” Journal of Virology 96, no. 20 (2022): 0131822, 10.1128/jvi.01318-22. PMC9599266 36173190

[26] Z. Bo and X. Li , “A Review of Pseudorabies Virus Variants: Genomics, Vaccination, Transmission, and Zoonotic Potential,” Viruses 14, no. 5 (2022): 1003, 10.3390/v14051003. 35632745 PMC9144770

[27] Q. Liu , Y. Kuang , Y. Li , et al., “The Epidemiology and Variation in Pseudorabies Virus: A Continuing Challenge to Pigs and Humans,” Viruses 14, no. 7 (2022): 1463, 10.3390/v14071463. 35891443 PMC9325097

[28] Q. Liu , X. Wang , C. Xie , et al., “A Novel Human Acute Encephalitis Caused by Pseudorabies Virus Variant Strain,” Clinical Infectious Diseases 73, no. 11 (2021): e3690–e3700, 10.1093/cid/ciaa987. 32667972

[29] Y. D. Tang , Y. Li , X. H. Cai , and X. Yin , “Viral Live‐Attenuated Vaccines (LAVs): Past and Future Directions,” Advanced Science 12, no. 3 (2025): 2407241, 10.1002/advs.202407241. 39639853 PMC11744563

[30] Y. D. Tang , C. Yu , and X. H. Cai , “Novel Technologies Are Turning a Dream into Reality: Conditionally Replicating Viruses as Vaccines,” Trends in Microbiology 32, no. 3 (2024): 292–301, 10.1016/j.tim.2023.09.002. 37798168

[31] Y. Q. Guo , M. H. Wang , N. Tang , et al., “Antimycin A Inhibits Alpha‐Herpesvirus Replication by Disrupting the Formation of Pyrimidinosomes,” Journal of Advanced Research (2025), 10.1016/j.jare.2025.05.016. PMC12869216 40354935

[32] T. Zhang , Y. Liu , Y. Chen , et al., “A Single Dose Glycoprotein D‐Based Subunit Vaccine Against Pseudorabies Virus Infection,” Vaccine 38, no. 39 (2020): 6153–6161, 10.1016/j.vaccine.2020.07.025. 32741670

[33] W. Mothes , N. M. Sherer , J. Jin , and P. Zhong , “Virus Cell‐to‐Cell Transmission,” Journal of Virology 84, no. 17 (2010): 8360–8368, 10.1128/JVI.00443-10. 20375157 PMC2918988

[34] L. E. Pomeranz , A. E. Reynolds , and C. J. Hengartner , “Molecular Biology of Pseudorabies Virus: Impact on Neurovirology and Veterinary Medicine,” Microbiology and Molecular Biology Reviews 69, no. 3 (2005): 462–500, 10.1128/MMBR.69.3.462-500.2005. 16148307 PMC1197806

[35] L. Na , Y. Zheng , J. B. Yang , H. L. Bao , and Y. D. Tang , “The Neonatal Fc Receptor (FcRn): Guardian or Trojan Horse in Viral Infection?,” PLOS Pathogens 21, no. 7 (2025): 1013285, 10.1371/journal.ppat.1013285. PMC12233286 40623027

[36] D. C. Roopenian and S. Akilesh , “FcRn: The Neonatal Fc Receptor Comes of Age,” Nature Reviews Immunology 7, no. 9 (2007): 715–725, 10.1038/nri2155. 17703228

[37] J. T. Sockolosky and F. C. Szoka , “The Neonatal Fc Receptor, FcRn, as a Target for Drug Delivery and Therapy,” Advanced Drug Delivery Reviews 91 (2015): 109–124, 10.1016/j.addr.2015.02.005. 25703189 PMC4544678

[38] M. Pyzik , L. K. Kozicky , A. K. Gandhi , and R. S. Blumberg , “The Therapeutic Age of the Neonatal Fc Receptor,” Nature Reviews Immunology 23, no. 7 (2023): 415–432, 10.1038/s41577-022-00821-1. PMC9891766 36726033

[39] D. Yang , M. Yang , S. Guo , et al., “Transition of Pseudorabies Virus from Latency to Reactivation State Selectively Triggered by Pathogenic Bacteria,” Science Advances 11 (2025): adw4206, 10.1126/sciadv.adw4206. PMC12629200 41259522

[40] Y. D. Tang , J. C. Guo , T. Y. Wang , et al., “CRISPR/Cas9‐Mediated 2‐sgRNA Cleavage Facilitates Pseudorabies Virus Editing,” The FASEB Journal 32, no. 8 (2018): 4293–4301, 10.1096/fj.201701129R. 29509513

[41] Y. Z. Bai , S. Wang , Y. Sun , et al., “The Full‐Length nsp2 Replicase Contributes to Viral Assembly in Highly Pathogenic PRRSV‐2,” Journal of Virology 99, no. 1 (2025): 0182124, 10.1128/jvi.01821-24. PMC11784222 39601570

[42] Y. Sun , L. Liu , H. Qiang , et al., “A Potent Broad‐Spectrum Neutralizing Antibody Targeting a Conserved Region of the Prefusion RSV F Protein,” Nature Communications 15, no. 1 (2024): 10085, 10.1038/s41467-024-54384-x. PMC11582626 39572535

[43] J. Hu , H. Tan , M. Wang , et al., “A Potent Protective Bispecific Nanobody Targeting Herpes Simplex Virus gD Reveals Vulnerable Epitope for Neutralizing,” Nature Communications 16, no. 1 (2025): 4196, 10.1038/s41467-025-58669-7. PMC12055985 40328740

[44] T. Y. Wang , F. D. Meng , G. J. Sang , et al., “A Novel Viral Vaccine Platform Based on Engineered Transfer RNA,” Emerging Microbes & Infections 12, no. 1 (2023): 2157339, 10.1080/22221751.2022.2157339. 36482724 PMC9769134

[45] T. Y. Wang , G. J. Sang , Q. Wang , et al., “Generation of Premature Termination Codon (PTC)‐Harboring Pseudorabies Virus (PRV) via Genetic Code Expansion Technology,” Viruses 14, no. 3 (2022): 572, 10.3390/v14030572. 35336979 PMC8950157

[46] Y. L. Yang , F. Meng , P. Qin , G. Herrler , Y. W. Huang , and Y. D. Tang , “Trypsin Promotes Porcine Deltacoronavirus Mediating Cell‐to‐Cell Fusion in a Cell Type‐Dependent Manner,” Emerging Microbes & Infections 9, no. 1 (2020): 457–468, 10.1080/22221751.2020.1730245. 32090689 PMC7054919

[47] Y. Y. Zhang , R. Liang , S. J. Wang , et al., “SARS‐CoV‐2 Hijacks Macropinocytosis to Facilitate Its Entry and Promote Viral Spike–Mediated Cell‐to‐Cell Fusion,” Journal of Biological Chemistry 298, no. 11 (2022): 102511, 10.1016/j.jbc.2022.102511. 36259516 PMC9484108

[48] H. L. Zhang , Y. M. Li , J. Sun , et al., “Evaluating Angiotensin‐Converting Enzyme 2‐Mediated SARS‐CoV‐2 Entry Across Species,” Journal of Biological Chemistry (2021): 296, 10.1016/j.jbc.2021.100435. PMC7892319 33610551

[49] T. Y. Wang , Y. L. Yang , C. Feng , et al., “Pseudorabies Virus UL24 Abrogates Tumor Necrosis Factor Alpha‐Induced NF‐κB Activation by Degrading P65,” Viruses 12, no. 1 (2020): 51, 10.3390/v12010051. 31906441 PMC7020041

