# Supplementary material for: A Humanized Anti‐gD Broadly Neutralizing Antibody Confers Complete Post‐Exposure Protection Against Pseudorabies Virus
Source: Adv Sci (Weinh). 2026 May 19;13(46):e75771. doi: 10.1002/advs.75771 (PMC13335967; doi:10.1002/advs.75771)
Supplement: Supplementary file 1 [file ADVS-13-e75771-s001.docx]

**Supporting Information**

**Figure S1.** **Assessment of the broad reactivity of mAb 6F7 against various PRV strains.** Vero-E6 cells were infected with PRV strains HeN1, TJ, SC, or Bartha K61 (MOI=0.01). Upon observation of significant cytopathic effect (CPE), cells were fixed, permeabilized, and blocked. Cells were incubated with mAb 6F7, followed by an FITC-conjugated goat anti-mouse IgG secondary antibody. Nuclei were counterstained with DAPI. Fluorescence was visualized using an inverted fluorescence microscope. The experiment was repeated three times, and a representative image is shown. Scale bars, 400 µm.

**Figure S2. mAb 6F7 does not inhibit PRV attachment to susceptible cells.** (**A**) Quantification of cell-associated viral DNA following attachment in PK15 cells. mAb 6F7 or an isotype control antibody (0.1, 1, or 10 µg) was pre-incubated with PRV HeN1 (at MOIs of 0.01, 0.1, or 1) for 2 h at 37°C. The mixtures were then added to pre-chilled PK15 cells and incubated at 4°C for 2 h to allow attachment. Unbound virus was removed by washing with PBS or (**B**) citric acid, and cell-associated viral DNA was quantified by qRT-PCR. (**C**) mAb 6F7 or an isotype control antibody (0.1, 1, or 10 µg) was pre-incubated with PRV HeN1 (at MOIs of 0.01, 0.1, or 1) for 2 h at 37°C. The mixtures were then added to pre-chilled Vero-E6, (**D**) HEK293T cells and incubated at 4°C for 2 h to allow attachment. Unbound virus was removed by washing with PBS, and cell-associated viral DNA was quantified by qRT-PCR. The experiment was repeated three times, and the results are presented as mean ± SD.

**Figure S3. Validation of FITC-conjugated anti-gB mAb.** Vero-E6 cells infected with PRV HeN1 (MOI=0.01) were fixed, permeabilized, and blocked upon CPE appearance. Cells were incubated with FITC-conjugated anti-gB mAb at the indicated dilutions (1:100, 1:500, 1:1000). Nuclei were stained with DAPI. Staining was assessed by fluorescence microscopy. The experiment was repeated three times, and a representative image is shown. Scale bars, 400 µm.

**Figure S4.** **mAb 6F7 does not affect PRV internalization in susceptible cells.** (**A**) Quantification of internalized viral DNA in PK15 cells. The mAb 6F7 or control mAb (0.1, 1, or 10 µg) was pre-incubated with PRV HeN1 (at MOIs of 0.01, 0.1, or 1) for 2 h at 37°C. Mixtures were added to pre-chilled PK15 cells and incubated at 4°C for 2 h for attachment. The inoculum was then removed, cells were washed with PBS or (**B**) citric acid, fresh pre-warmed medium was added, and cells were shifted to 37°C for 2 h to permit internalization. After washing, cells were harvested, and internalized viral DNA was quantified by qRT-PCR. (**C**) The mAb 6F7 or control mAb (0.1, 1, or 10 µg) was pre-incubated with PRV HeN1 (at MOIs of 0.01, 0.1, or 1) for 2 h at 37°C. Mixtures were added to pre-chilled Vero-E6, (**D**) HEK293T cells and incubated at 4°C for 2 h for attachment. The inoculum was then removed, cells were washed with PBS, fresh pre-warmed medium was added, and cells were shifted to 37°C for 2 h to permit internalization. After washing, cells were harvested, and internalized viral DNA was quantified by qRT-PCR. The experiment was repeated three times, and the results are presented as mean ± SD.

**Figure S5. Validation of FITC-conjugated mAb 6F7 (anti-gD).** Vero-E6 cells infected with PRV HeN1 (MOI=0.01) were processed as in Figure S3. Cells were incubated with FITC-conjugated mAb 6F7 (anti-gD) at the indicated dilutions (1:100, 1:500, 1:1000) and visualized. The experiment was repeated three times, and a representative image is shown. Scale bars, 400 µm.

**Figure S6. Requirement of gD for viral glycoprotein-mediated cell fusion.** (**A**) RK13 cells in 24-well plate were co-transfected with plasmids expressing EGFP (20 ng), gB, gH, gL (100 ng each), and either gD or an empty control plasmid (100 ng). After 36 h, cells were stained with DAPI and observed for syncytia formation via fluorescence microscopy. The experiment was repeated three times, and a representative image is shown. (**B**) The relative fusion area was quantified based on EGFP fluorescence using ImageJ. The fusion area in cells transfected with the full complement of glycoproteins (gB, gD, gH, gL) was set to 100%. The experiment was repeated three times, and the results are presented as mean ± SD.

**Figure S7.** **Comparison of the predicted gD-Nectin-1 complex with the experimentally resolved crystal structure of gD-SW-Nectin-1.** The superimposition of the AlphaFold 3‑predicted gD–Nectin‑1 complex model (gD shown in pink, Nectin‑1 in light blue) with the experimentally resolved crystal structure of the gD–SW–Nectin‑1 complex reported in the literature (gD in light purple, Nectin‑1 in orange).

**Figure S8. mAb 6F7 cannot neutralize HSV-1.** (**A**) HSV-1-EGFP strains (200 TCID_50_) was incubated with 50 µL of indicated concentrations of mAb 6F7 or mAb control for 2 h at 37°C. The mixtures were then transferred to a 96-well plate containing a monolayer of Vero-E6 cells and incubated for an additional 2 h at 37°C. After removal of the inoculum, cells were washed and cultured in fresh medium containing 2% FBS. At 36 h post-infection, infected cells were examined under a microscope. The experiment was repeated three times, and a representative image is shown. Scale bars, 400 µm. (**B**) The relative infection was determined by quantifying the area occupied by EGFP-positive cells using ImageJ. The EGFP area in the PBS control was set to 1.0. The experiment was repeated three times, and the results are presented as mean ± SD.
